# Durable, superoleophobic polymer–nanoparticle composite surfaces with re-entrant geometry via solvent-induced phase transformation

**DOI:** 10.1038/srep21048

**Published:** 2016-02-15

**Authors:** Philip S. Brown, Bharat Bhushan

**Affiliations:** 1 Nanoprobe Laboratory for Bio- & Nanotechnology and Biomimetics (NLBB) The Ohio State University, 201 W. 19th Avenue Columbus, OH 43210-1142, USA

## Abstract

Superoleophobic plastic surfaces are useful in a wide variety of applications including anti-fouling, self-cleaning, anti-smudge, and low-drag. Existing examples of superoleophobic surfaces typically rely on poorly adhered coatings or delicate surface structures, resulting in poor mechanical durability. Here, we report a facile method for creating re-entrant geometries desirable for superoleophobicity via entrapment of nanoparticles in polycarbonate surfaces. Nanoparticle incorporation occurs during solvent-induced swelling and subsequent crystallization of the polymer surface. The resulting surface was found to comprise of re-entrant structures, a result of the nanoparticle agglomerates acting as nucleation points for polymer crystallization. Examples of such surfaces were further functionalized with fluorosilane to result in a durable, super-repellent surface. This method of impregnating nanoparticles into polymer surfaces could prove useful in improving the anti-bacterial, mechanical, and liquid-repellent properties of plastic devices.

Oil repellency (superoleophobicity) is a desirable surface property for a range of different applications including anti-fouling^1^, self-cleaning^2^, anti-smudge^3^,^4^, and lab-on-chip^5^ applications. However, superoleophobicity is difficult to accomplish as the surface tensions of oils are much lower than that of water, meaning oil droplets are more likely to display contact angles of <90° on flat surfaces and therefore adding roughness to the surface will lower this angle further.

However, high droplet contact angles can still be achieved, even if the contact angle on the flat surface (*θ*_*flat*_) is low, through the use of re-entrant geometries, where surface asperities create an overhang^6^ (i.e. become narrower closer to the surface), Fig. 1. For example, for a surface with inverse trapezoidal features, if the combination of the re-entrant angle (α) and *θ*_*flat*_ is ≥90°, the geometry is able to support a favorable shape for the liquid–vapor interface (surface tension is pointing upward) and the liquid does not fully wet the surface (vapor pockets and a composite interface with a low liquid-solid contact fraction), Fig. 1a.

Structures with re-entrant curvature (spherical, cylindrical, oval etc.) as shown in Fig. 1b are able to support high droplet contact angles for various liquids with flat contact angles <90° since it is possible to draw multiple tangents of a corresponding flat surface, as demonstrated in Fig. 1c. Therefore liquids with various flat contact angles can wet the re-entrant curvature to different extents to achieve a favorable liquid–vapor interface shape. The re-entrant structures in Fig. 1d are able to support low flat contact angles (0° < *θ*_*flat*_ < 90°) and, while they could theoretically support flat contact angles of 0°, the presence of positive pressure in the droplet means that a liquid-vapor interface shape with upward surface tension is required. This is possible with doubly re-entrant geometries, as shown in Fig. 1e, which incorporate vertical overhangs normal to the surface in addition to the horizontal overhangs parallel to the surface found in Fig. 1d. Here, tangents on the re-entrant curvature can be drawn beyond those parallel to the flat surface and so a favorable liquid–vapor interface shape where the surface tension is pointing upward can be supported, even with a fully wetting liquid^7^. Such structures need to be designed to minimize the liquid-solid contact fraction to ensure the droplet is resting mostly on air pockets for the surface to be repellent. The structures therefore need to be as thin as possible^7^. However, such surface features are fragile and difficult to fabricate on a large scale and therefore singly re-entrant geometries are more commonly used in liquid repellency.

To repel liquids with low surface tensions, these re-entrant geometries typically need to be paired with a low surface energy material^8^,^9^. Fluorinated materials are commonly used as fluorine is very electronegative and has a low polarizability. This results in a low susceptibility to London dispersion forces, leading to weak intermolecular forces, weak cohesive and adhesive forces, and therefore low surface energies.

Many existing methods for creating superoleophobic surfaces rely on coatings to add roughness and low surface tension material to a substrate^3^,^4^. Several use a “one-pot” technique with all materials mixed and deposited together^10^,^11^. However such techniques typically suffer from poor durability due to the weak interfacial adhesion between the substrate and the low surface tension material required for oil repellency. Instead of using a coating, it is therefore often desirable to treat the surface of the substrate directly to add roughness and lower the surface tension. Techniques specific to different substrates therefore need to be developed. For example, acid etching has been shown to be an effective way of creating superoleophobic aluminum surfaces^12^.

Water and oil-repellent plastic surfaces are of interest for a wide range of industrial and consumer applications such as the creation of packaging that is not fouled by product, reducing wastage and improving the customer experience. Existing methods for creating nanotextured polymer surfaces typically rely on techniques such as plasma etching^13^, or photolithography^14^, top-down approaches that are expensive, time-consuming and result in wasted material^15^. Alternatively, the roughness of a polymer surface can be altered through the use of nanoparticles. This is sometimes achieved by adding nanomaterials to the polymer melt, however this can result in varying degrees of particle dispersion and the ideal nanocomposite morphology not being met^16^. Nanoobjects can also be dispersed in a monomer, which is then polymerized *in situ,* allowing for better dispersion of the nanomaterial and less agglomeration, however this technique relies on the use of organic solvents and is less compatible with common industrial processes such as extrusion^17^. Another method to add nanoparticles is to use supercritical CO_2_ to impregnate the polymer^18^, however this requires the use of high-pressure equipment^19^ and results in nanoparticles being impregnated throughout the plastic, affecting the properties of the bulk.

In addition to liquid repellency, polymer nanocomposites find use in other applications. For instance, silver nanoparticles have previously been impregnated into polycarbonate to improve the anti-bacterial properties of plastic catheters^20^,^21^. Zirconia nanoparticles were added to polymer films to result in an ultra-hard coating^22^. Finally, gold nanoparticles draw substantial interest due to their catalytic activity and polymer-gold nanocomposites have been shown to catalyze organic reactions^23^. In the above examples, the presence of nanoobjects at the surface or near surface gave rise to the desired properties. Similarly for liquid repellency, it is desirable for the nanoparticle agglomerates to be present at the interface to provide the re-entrant geometry required.

In this paper, durable superoleophobic polycarbonate has been created through the incorporation of nanoparticles into the polymer surface. Polycarbonate undergoes solvent-induced phase transformation when exposed to acetone and the resulting crystallization leads to a rough, superhydrophobic surface. By introducing nanoparticles into the acetone solvent, polycarbonate–nanoparticle (PC–NP) composites surfaces with re-entrant geometry have been created. The durability and functionality of the surfaces has been tested; durability is important if these surfaces are to be feasible for application in various industries including medical, transportation, aerospace, energy, and construction. Finally, since the nanoparticles are only incorporated near the interface, this technique could be advantageous compared to other polymer nanocomposite fabrication techniques where nanoparticles are distributed throughout the polymer, affecting the properties of the bulk material.

## Results and Discussion

**Figure 2:**
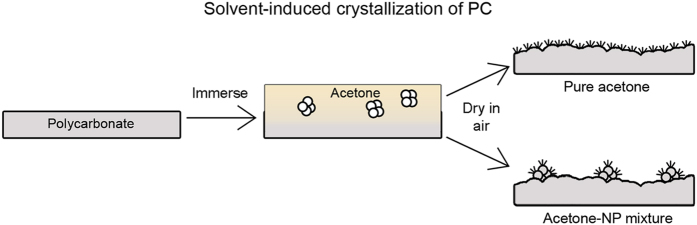
Schematic of solvent-induced crystallization of polycarbonate using either pure acetone or an acetone–NP mixture.

In order to achieve re-entrant structures amenable to creating a superoleophobic surface, the surfaces described in this paper comprise polycarbonate treated with an acetone–nanoparticle (acetone–NP) mixture. It is found that the nanoparticles become incorporated into the polymer during swelling and the subsequent polymer crystallization can be directed, Fig. 2. By creating polymer–NP composite surfaces in this way, re-entrant geometries are achieved as the nanoparticle agglomerates are impregnated into the surface and near-surface region of the polymer. Nanoparticles incorporated deeper into the polymer would have a less pronounced effect on the surface topography. Small, hydrophilic SiO_2_ nanoparticles were used as these have been shown to be susceptible to irreversible aggregation^24^, resulting in non-spherical, micron-sized clusters. In addition, SiO_2_ nanoparticles are known to have high hardness^25^, which will aid in the creation of a mechanically durable coating^26^. To achieve the oil repellency, the polymer-nanoparticle composite surface was then activated using UV irradiation and treated with fluorosilane. The re-entrant surface roughness due to nanoparticle incorporation and solvent-induced phase transformation enhances the surface properties of the fluorosilane to result in a superoleophobic surface.

### Wettability of surfaces

**Figure 3:**
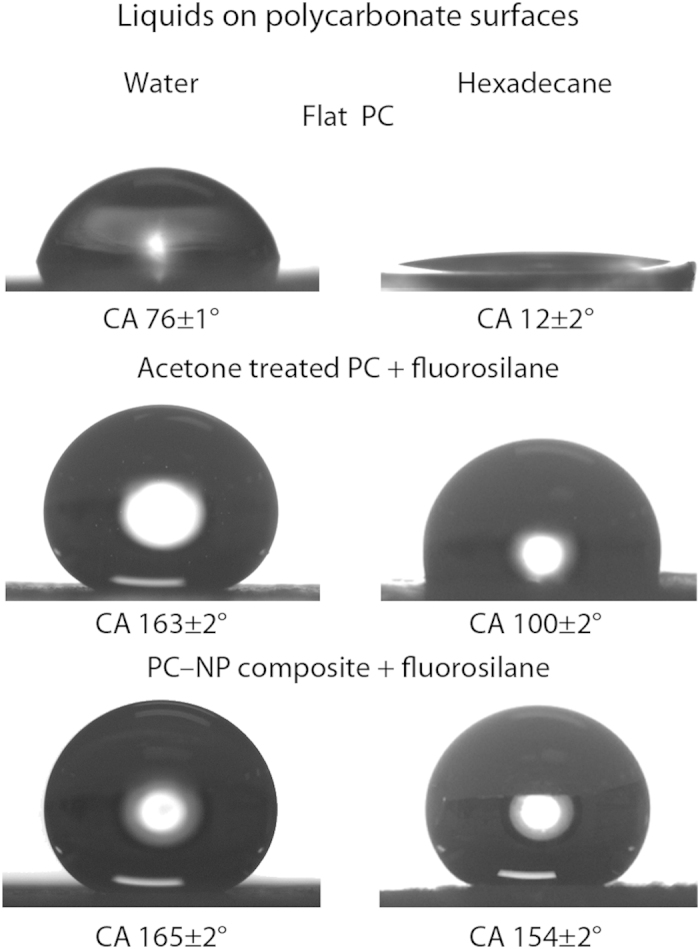
Contact angle images for droplets water and hexadecane on: flat PC; fluorinated, acetone-treated PC; and fluorinated PC–NP composite surfaces.

**Figure 4:**
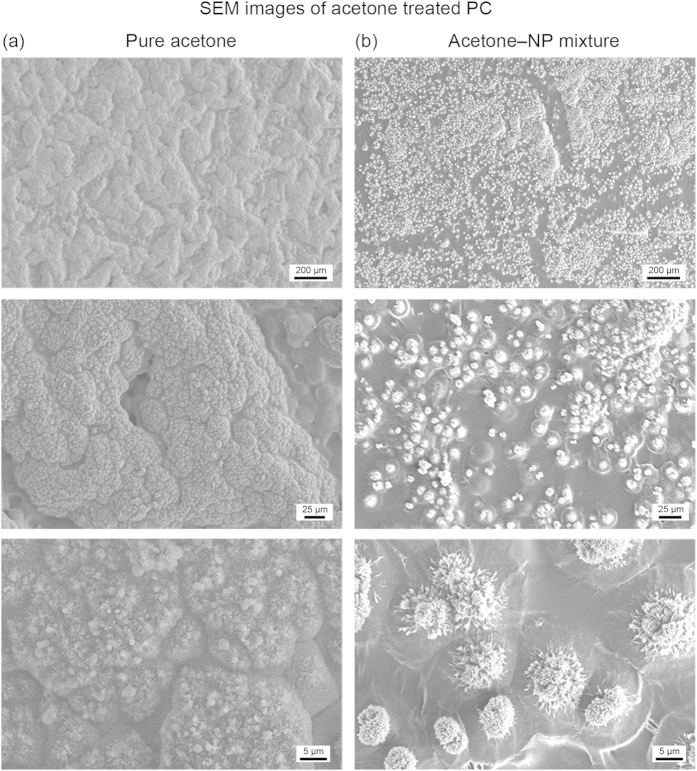
SEM images of solvent-induced crystallization of polycarbonate using either pure aceteone or an optimized acetone–NP mixture. (**a**) For pure acetone, the resulting surface contains good coverage of nanostructures atop micron-sized mounds. (**b**) For the actone–NP mixture, the nanostructures are limited to discrete micron-sized spherulites due to the nanoparticle aggregates acting as nucleation sites for the polycarbonate crystallization.

Untreated polycarbonate (flat PC) is found to be slightly hydrophilic with water contact angles of 76 ± 1°, Table 1 and Fig. 3. After immersion in acetone for 5 min followed by drying in air, the surface became opaque; suggesting crystallization of the polymer had taken place. Previous studies have confirmed that the crystallinity increases after acetone treatment^27^. Indeed, the polycarbonate undergoes a solvent-induced phase transformation leading to a hierarchical structure of crystalline nanostructures atop micron-sized mounds, Fig. 4(a). This roughness is sufficient to create a superhydrophobic surface with water droplets displaying contact angles of 158 ± 1° with low tilt angles due to the ability of the hierarchical surface to trap air and thus create a composite interface. However, due to the low contact angles for hexadecane on flat polycarbonate (ca. 12°), the acetone-treated surface is superoleophilic with hexadecane fully wetting the surface. Activation of the surface by UV irradiation followed by fluorination via silane attachment leads to hexadecane angles of 76 ± 2° for the flat surface and 100 ± 2° for the acetone-treated surface, Fig. 3. This low angle is due to the lower surface tension of hexadecane being unable to support a composite interface on the hierarchical structure of the acetone-treated polycarbonate.

**Figure 5:**
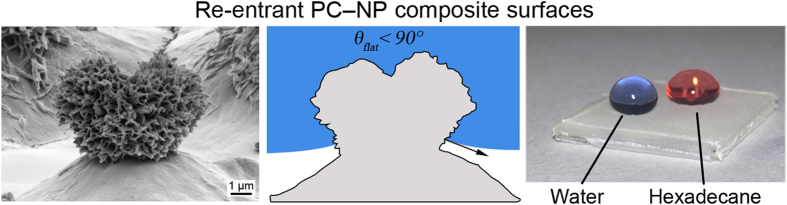
SEM images of the PC–NP composite surface displaying re-entrant geometry. This re-entrant surface, once fluorinated, was found to be repellent towards both hexadecane and water.

In order to create superoleophobic surfaces, re-entrant geometries must be incorporated into the polycarbonate. To accomplish this, nanoparticles were added to the acetone solvent used during the solvent-induced phase transformation of polycarbonate. Polycarbonate samples were placed in the acetone–NP mixture for 5 min and then air-dried. The resulting surface is found to be dramatically different from that of the acetone-treated polycarbonate. Instead of a consistent coverage of nanostructures atop micron mounds, the nanotexturing is instead limited to discrete micron-sized spherulites, Fig. 4(b). This is believed to be due to the presence of the nanoparticle agglomerates, which are incorporated into the surface and near-surface during polymer swelling and act as nucleation sites for polycarbonate crystallization during evaporation of the solvent^28^. The result is re-entrant, hierarchical structures as shown in Fig. 5.

**Figure 6:**
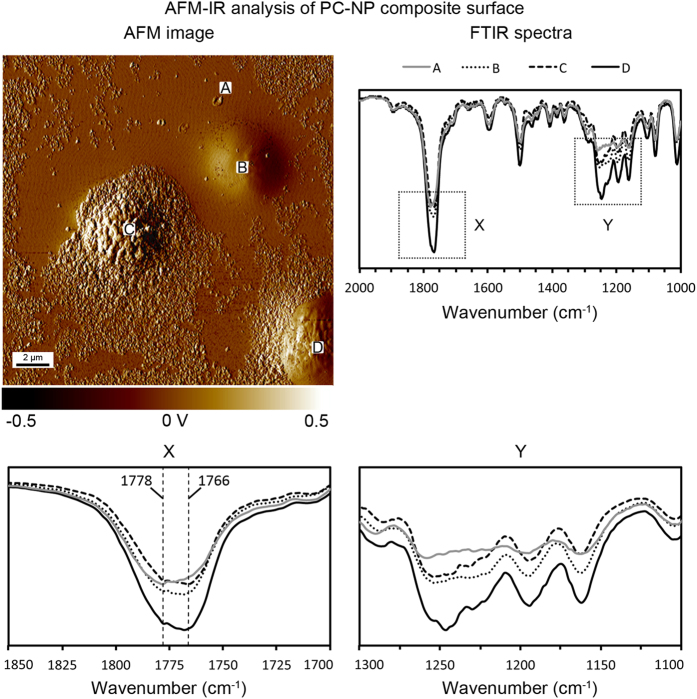
AFM-IR analysis of PC–NP composite surface. The AFM map displays the untextured region (**A**) and the re-entrant hierarchical structures (**B–D**) selected for IR analysis. The FTIR spectra confirm that polycarbonate is present in all regions and that the polymer crystallinity increases on the re-entrant hierarchical structures, suggesting solvent-induced polymer recrystallization occurs primarily on the nanoparticle agglomerates.

Combined AFM-IR measurements were used to further analyze the polycarbonate-nanoparticle (PC–NP) composite surface, and confirmed that the nanoparticle agglomerates become embedded into the polymer surface, Fig. 6. Infrared bands characteristic of polycarbonate were present on both the untextured surface (region A) and the re-entrant hierarchical structures (regions B–D). The major infrared peak classifications were found to be as follows^29^ C = O stretch (1775 cm^−1^), C = C stretch (1496 cm^−1^), and C-O-C bands (1300–1100 cm^−1^). Further analysis of the carbonyl stretch revealed that it consists of two peaks at 1778 and 1766 cm^−1^, the relative intensities of which are known to reflect the amount of non-crystalline and crystalline polycarbonate respectively^30^. By comparing the infrared spectra from the untextured region with that from the re-entrant hierarchical structures, it was found that the relative intensity of the 1766 cm^−1^ band increased, suggesting an increase in crystallinity. Sharper, more defined peaks from the re-entrant hierarchical structures compared to the untextured polymer also suggest an increase in crystallinity. This supports the theory that solvent-induced recrystallization of the polycarbonate occurs predominantly at the nanoparticle agglomerates, likely due to the agglomerates acting as nucleation points.

The water contact angle of the polycarbonate-nanoparticle (PC–NP) composite surface is 30 ± 2°. After UV activation (to facilitate silane attachment) and fluorosilane treatment, the surface becomes superhydrophobic and superoleophobic with contact angles of 165 ± 2° and 154 ± 2° for water and hexadecane respectively, Fig. 3. The presence of the re-entrant, hierarchical structures enables the formation of a favorable liquid-vapor interface for lower surface tension liquids, such as hexadecane, that exhibit contact angles <90° on the corresponding flat surface.

**Figure 7:**
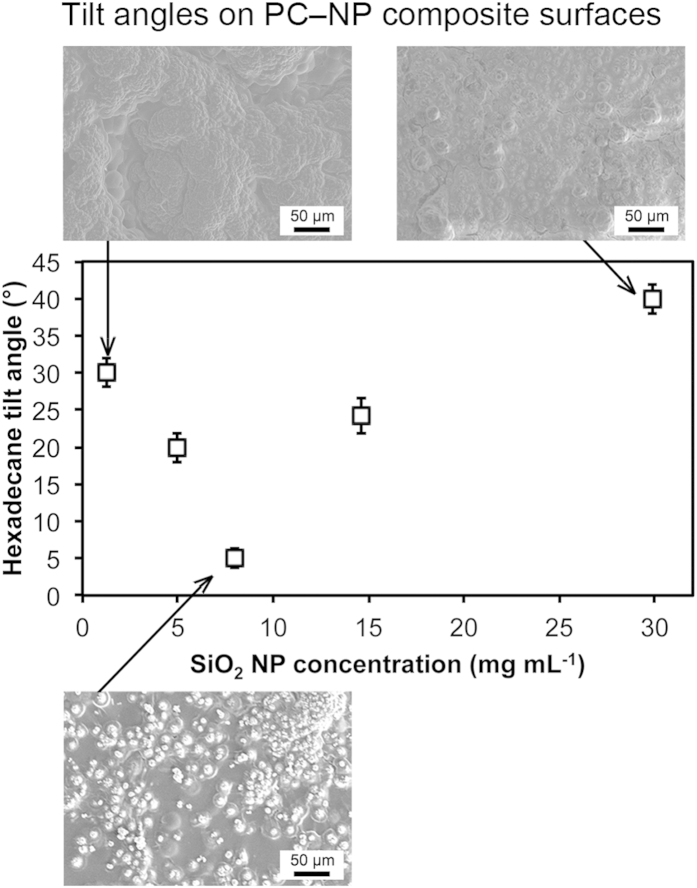
Graph to show hexadecane tilt angles on fluorinated PC–NP composite surfaces as a function of SiO_2_ NP concentration used during acetone–NP treatment. Inset: SEM images of PC–NP composite surfaces treated with acetone–NP mixtures of varying SiO_2_ NP concentrations.

In order to optimize the surface, the concentration of nanoparticles in the acetone solvent was varied. The hexadecane tilt angle was used as a measure of optimizing the surface treatment, Fig. 7. SEM images were taken of the surfaces formed after treatment with various concentrations of nanoparticle-acetone mixtures. At low nanoparticle concentrations, it was found that the surface closely resembles that of the pure acetone treated surface shown in Fig. 4(a). It is theorized that the nanoparticle concentration is too low for the resulting nanoparticle agglomerates to form nucleation points for the polymer crystallization upon acetone evaporation, the result is poor oil repellency and a high hexadecane tilt angle. As the nanoparticle concentration is increased, discrete spherulites begin to form and the re-entrant geometry of these spherulites results in good oil-repellency and a reduced hexadecane tilt angle. An optimum hexadecane tilt angle of 5° was found on the fluorinated PC–NP composite surface resulting from an acetone–NP mixture concentration of around 8 mg mL^−1^. Above this concentration and the hexadecane tilt angle begins to rise due to contact line pinning on the surface features. At high nanoparticle concentrations, the agglomerates are larger and no longer discrete enough to act as nucleation points for the polymer crystallization, which instead occurs across the entire surface, covering the agglomerates and the space between and resulting in a high hexadecane tilt angle.

### Wear resistance of surface

**Figure 8:**
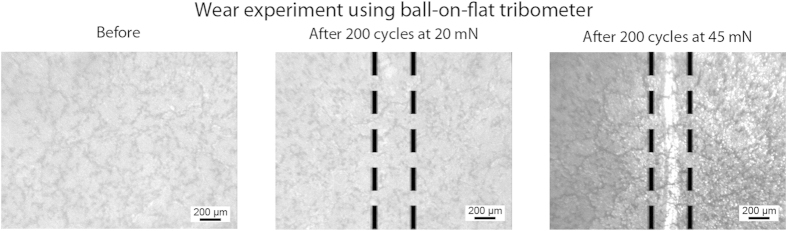
Optical micrographs before and after wear experiments using ball-on-flat tribometer using a 3-mm diameter sapphire ball at 20 mN and 45 mN loadings for fluorinated PC–NP composite surfaces.

The mechanical durability of the polymer-nanoparticle composite surface was investigated through the use of tribometer wear experiments and the resulting optical images, showing a portion of the wear track, are displayed in Fig. 8. The initial wear experiments were carried out with a load of 20 mN, however few observable defects were found on the surface after this experiment and the coating remained oil repellent (tilt angle remains 5 ± 2°), suggesting good fluorosilane attachment to the polymer. To further test the durability of the surface, the load was increased to 45 mN. This increased load resulted in an observable wear scar, upon which hexadecane droplets were found to pin (tilt angle increases to ca. 20°) when rolled over the wear scar location. However higher magnification images confirmed that the surface features were not completely destroyed and, after re-application of the fluorosilane, the surface was found to regain its oil repellency (tilt angle 5 ± 2°) suggesting that only the low surface tension material was removed at the higher load and that any damage to the underlying polymer–nanoparticle composite structures was minimal. It is believed that these surfaces are significantly more durable than current examples of superoleophobic surfaces, which typically rely on poorly adhered coatings or delicate surface structures, studies for which typically report either poor mechanical properties or fail to report any durability data. In addition, these surfaces were found to maintain their repellency towards water and oil after 6 months of storage with no noticeable degradation in their liquid repellent properties.

### Self-cleaning property of coated samples

**Figure 9:**
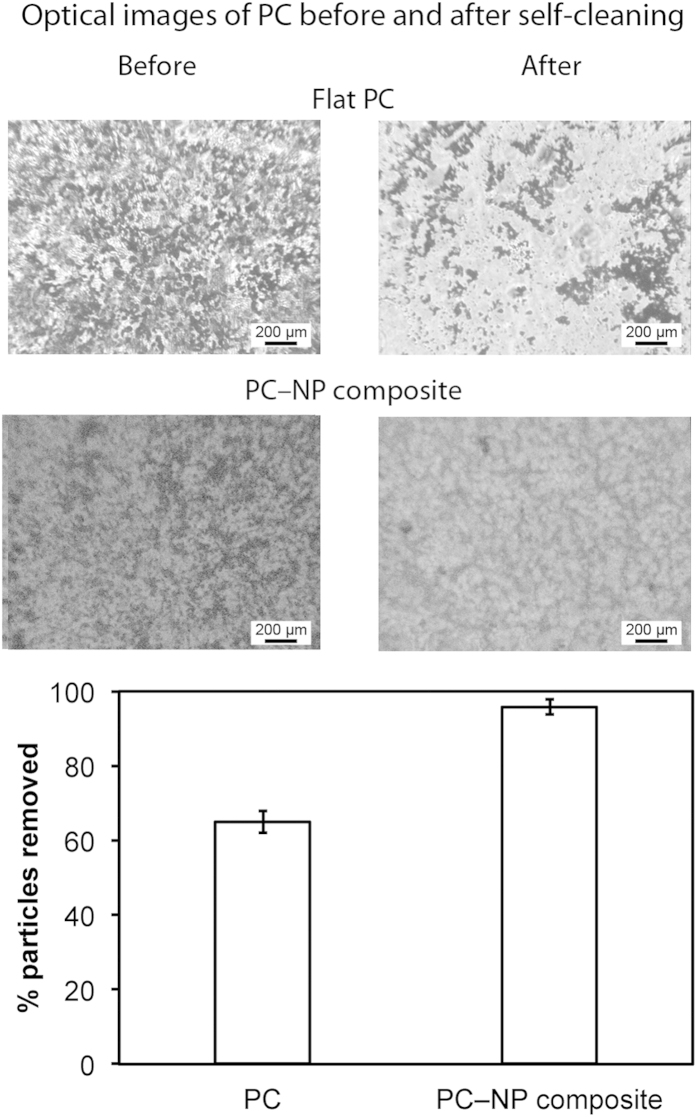
Optical micrographs of contaminated coatings before and after self-cleaning test on flat PC and fluorinated PC–NP composite surfaces. Dark spots on coatings and cloth indicate silicon carbide particle contaminants. Image analysis suggests a >90% removal of particles on the composite surface.

To examine the self-cleaning properties, the coatings were contaminated with silicon carbide particles, shown in Fig. 9. A stream of water droplets was then used to clean the surface. On the flat PC coating this resulted in an incomplete removal of the particles with the surface remaining contaminated whereas for the PC–NP composite surface, the water droplets removed almost all of the particles. The superhydrophobic surface is self-cleaning due to the high contact angle and low hysteresis for water. Droplets of water deposited onto this sample are able to roll over the surface with little impediment, collecting less hydrophobic contaminants as they go.

### Anti-smudge property of coated samples

**Figure 10:**
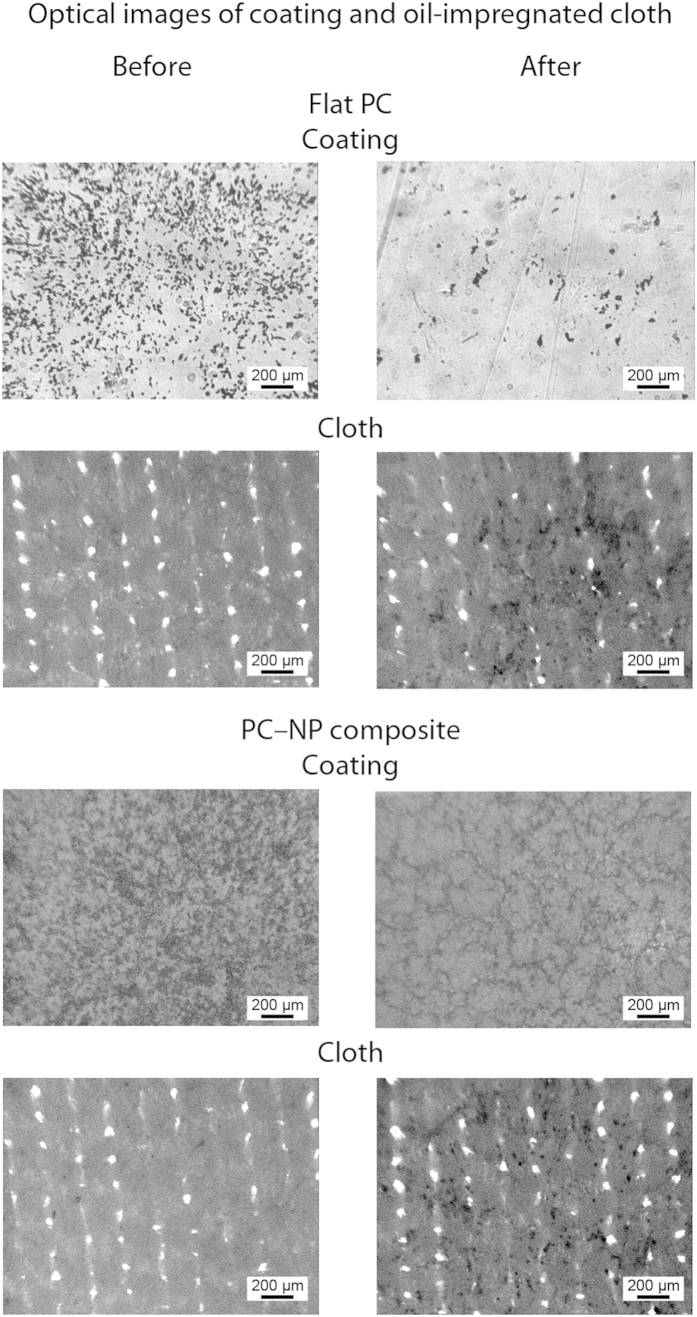
Optical micrographs of contaminated coatings and oil-impregnated microfiber cloth before and after smudge test on flat PC and fluorinated PC–NP composite surfaces. Dark spots on coatings and cloth indicate silicon carbide particle contaminants.

To examine the anti-smudge properties of the PC–NP composite surfaces a hexadecane-soaked cloth was used to wipe the contaminated surfaces, shown in Fig. 10. On the flat PC surface this resulted in incomplete removal of the particles with the surface remaining contaminated. For the oil-repellent PC–NP composite surface, the particles were transferred to the cloth with no observable particles remaining on the surface. Similarly to the self-cleaning experiments with water, the anti-smudge property relies on a high contact angle and low hysteresis for the oil. The oil in the cloth is able to collect oleophilic contaminants from the surface of the coating without sticking to the surface.

## Conclusions

Durable, superoleophobic plastic surfaces have been created through a facile method involving the impregnation of polycarbonate with silica nanoparticles during solvent-induced phase transformation. Following treatment, re-entrant structures are present on the surface due to the nanoparticle agglomerates acting as nucleation sites for polymer crystallization. After fluorosilane treatment, the surfaces displayed both high contact angles and low tilt angles for water and hexadecane. The super-repellent surfaces were found to be more durable compared to existing superoleophobic surfaces and coatings. The self-cleaning and anti-smudge properties of the surfaces were also demonstrated. It is further envisaged that this technique could be applied to other polymer–solvent combinations where solvent-induced phase transformation occurs. It is also believed that parameters such as nanoparticle size, shape, and chemistry as well as treatment time, temperature and drying conditions will effect the formation of the composite surfaces and therefore the resulting surface properties. Such incorporation of nanoparticles into existing polymer surfaces is an attractive way to improve the anti-bacterial, mechanical, and liquid-repellent properties of plastic devices.

## Methods

### Samples

Polycarbonate sheet (PC, Lexan 9030, SABIC Innovative Plastics IP BV) cut to dimensions of 25 by 10 mm were used throughout. Silica nanoparticles (NP, 7 nm diameter, Aerosil 380, Evonik Industries) were dispersed in acetone (Fisher Scientific Inc.) using an ultrasonic homogenizer (Branson Sonifier 450 A, 20 kHz frequency at 35% amplitude) at various concentrations. The polycarbonate samples were immersed in the acetone-NP mixture for 5 min before being removed and allowed to dry in air. To activate the polymer surface for silane attachment, samples were either UV irradiated for 40 min (15 W, λ_max_ = 254 nm) or treated with O_2_ plasma (Plasmalab System 100, Oxford Instruments) at an O_2_ flow rate of 20 sccm and a power of 40 W for 2 min. Both techniques were found to result in similar levels of activation and only UV irradiated samples are reported. Samples were fluorinated via chemical vapor deposition of a silane, which was required in order to achieve superoleophobicity. One drop of trichloro(1 H,1 H,2 H,2 H-perfluorooctyl) silane (fluorosilane, Sigma Aldrich) was deposited next to the samples which were covered and left for 6 h.

### Contact angle and tilt angle

For contact angle data, 5-μL droplets of water and n-hexadecane (99%, Alfa Aesar) were deposited onto samples using a standard automated goniometer (Model 290, Ramé-Hart Inc.) and the resulting image of the liquid–air interface analyzed with DROPimage software. Tilt angles were measured by inclining the surface until the 5-μL droplet rolled off. All angles were averaged over at least five measurements on different areas of a sample.

### Scanning electron microscope imaging

Top down, scanning electron microscope (SEM, Hitachi S-4300) images were taken to determine the topography of the polycarbonate samples. To image the re-entrant geometry, SEM images (Philips/FEI Sirion) were taken with the sample held at a 75° angle. Samples were mounted with conductive tape and gold-coated prior to SEM imaging.

### Atomic force microscopy-infrared measurements

AFM-IR measurements were carried out on a nanoIR-2 (Anasys Instruments) equipped with tunable optical parametric oscillator laser system (ELSPLA, Model# NT-277/3-XIR3-1 K, rep rate 1 kHz). The AFM images were captured using tapping mode NIR2 probes (PR-EX-TnIR-A-10, resonance frequency = 75 kHz, spring const. = 1–7 N m^−1^).

### Wear experiments

The mechanical durability of the surfaces was examined through macrowear experiments performed with an established procedure of using a ball-on-flat tribometer^26^. A sapphire ball of 3 mm diameter was fixed in a stationary holder. Loads of 20 mN and 45 mN were applied normal to the surface, and the tribometer was put into reciprocating motion for 200 cycles. Stroke length was 6 mm with an average linear speed of 1 mm s^−1^. Surfaces were imaged before and after the tribometer wear experiment using an optical microscope with a CCD camera (Nikon Optihot-2) to examine any changes^25^.

Contact pressures for the tribometer wear experiments were calculated based on Hertz analysis^26^. The elastic modulus of polycarbonate^31^, 2.3 GPa, was used as an estimate for the elastic modulus of the composite coating, and a Poisson’s ratio of 0.37 was used^32^. Due to the inclusion of SiO_2_ nanoparticles, the elastic modulus of final coating is expected to be higher, so an underestimated pressure will be obtained with the selected modulus. An elastic modulus of 390 GPa and Poisson’s ratio of 0.23 were used for sapphire ball used in the macroscale wear experiments^33^ and the mean contact pressures were calculated as 15.3 MPa and 20.0 MPa for low and high loads respectively.

### Self-cleaning experiment

The self-cleaning characteristics of the surfaces were examined using an experimental setup previously reported^4^. Coatings were contaminated with silicon carbide (SiC, Sigma Aldrich). The contaminated sample was then secured on a stage (45° tilt) and water droplets (total volume 5 mL) were dropped onto the surface from a specified height. The ability for the water stream to remove particles was quantified using image analysis software (SPIP 5.1.11, Image Metrology A/S, Horshølm, Denmark).

### Anti-smudge experiment

The anti-smudge characteristics of the surfaces were examined using an experimental setup previously reported^4^. A hexadecane-soaked microfiber cloth was set to rub the contaminated sample under a load of 5 g for 1.5 cm at a speed of about 0.2 mm s^−1^.

## Additional Information

**How to cite this article**: Brown, P. S. and Bhushan, B. Durable, superoleophobic polymer-nanoparticle composite surfaces with re-entrant geometry via solvent-induced phase transformation. *Sci. Rep.* **6**, 21048; doi: 10.1038/srep21048 (2016).

## Figures and Tables

**Figure 1:**
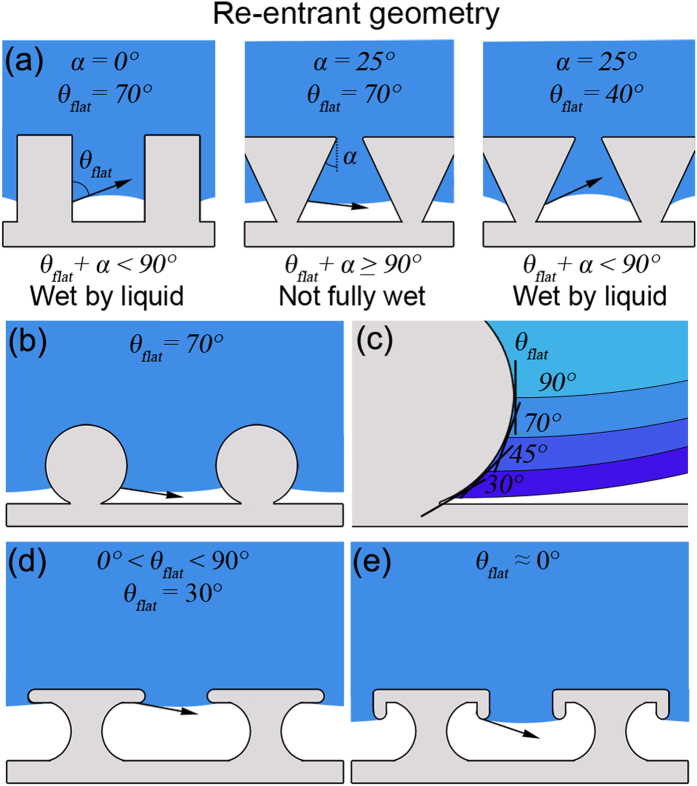
(**a**) *θ*_*flat*_ angles of <90° on non–re-entrant and re-entrant geometries, liquid does not fully wet structure if *θ*_*flat*_ + α ≥ 90° thanks to favorable shape of the liquid–vapor interface, (**b**) geometry with re-entrant curvature supporting a *θ*_*flat*_ angle 70°, (**c**) geometry with re-entrant curvature supporting various *θ*_*flat*_ angles of ≤90°, (d) re-entrant geometry supporting *θ*_*flat*_ angles of 30°, and (**e**) doubly re-entrant geometry supporting *θ*_*flat*_ angles of ≈0° 6.

**Table 1:** Comparison of static contact angles and tilt angles for water and hexadecane droplets deposited on polycarbonate surfaces.

Surface	Water		Hexadecane	
	Contact angle (°)	Tilt angle (°)	Contact angle (°)	Tilt angle (°)
Flat PC	76 ± 1	N/A	12 ± 2	N/A
Flat PC + fluorosilane	110 ± 2	N/A	76 ± 2	N/A
Acetone treated PC	158 ± 1	3 ± 1	~0	N/A
Acetone treated PC + fluorosilane	163 ± 2	2 ± 1	100 ± 2	N/A
PC–NP composite surface	30 ± 2	N/A	~0	N/A
PC–NP composite surface + fluorosilane	165 ± 2	2 ± 1	154 ± 2	5 ± 2
